# Survival Prognosis of Japanese With Severe Motor and Intellectual Disabilities Living in Public and Private Institutions Between 1961 and 2003

**DOI:** 10.2188/jea.JE20090024

**Published:** 2010-01-05

**Authors:** Tomoyuki Hanaoka, Katsumi Mita, Azuma Hiramoto, Yasuyuki Suzuki, Shizuo Maruyama, Toshio Nakadate, Reiko Kishi, Kitoku Okada, Yasuhiko Egusa

**Affiliations:** 1 Bihoro Ryoiku Hospital, Abashiri-gun, Hokkaido, Japan; 2 Seijoh University, Tokai, Aichi, Japan; 3 Hokkaido Ryoikuen, Asahikawa, Hokkaido, Japan; 4 Tokyo Children’s Rehabilitation Hospital, Musashimurayama, Tokyo, Japan; 5 Showa Medical University, Tokyo, Japan; 6 Hokkaido University, Sapporo, Japan; 7 Kawasaki University of Medical Welfare, Kurashiki, Okayama, Japan

**Keywords:** prognosis for survival, severe motor and intellectual disabilities, survival analysis

## Abstract

**Background:**

Although the prognosis for survival in people with severe functional disabilities is a serious concern for their families and health care practitioners, there have been few reports on survival rates for this population. Every year, the Japanese Association of Welfare for Persons with Severe Motor and Intellectual Disability collects anonymous records of individual registrations and deaths from all private and public institutions, excepting national institutions. We used these data to estimate the prognosis for survival.

**Methods:**

We reviewed the records of 3221 people with severe motor and intellectual disabilities (SMID); all subjects had lived in one of 119 public or private institutions in Japan between 1961 and 2003. Kaplan–Meier survival estimates were calculated according to disability type and birth year range.

**Results:**

Of the 3221 persons, 2645 were alive and 576 had died. The survival rate at the age of 20 for all subjects was 79% (95% confidence interval, 78%–81%). Among people who were unable to sit, those with lower intelligence quotients had lower survival rates.

**Conclusions:**

The survival rate among people with SMID housed in public and private institutions in Japan was much worse than that of the general population, and has not improved since the 1960s.

## INTRODUCTION

Before 1950, Japanese with severe functional disabilities lived with their families and had little chance to receive medical care. The first institution in Japan to provide care for people with severe functional disabilities was established by volunteers in 1961. In 1967, the concept of “severe motor and intellectual disabilities,” or SMID, and a system to build facilities to care for people with SMID, were legally established.^1^ According to the standard classification criteria (Oshima’s classification), a person with SMID is defined as one who is bedridden or able to sit, crawl, or walk with support, and has an intelligence quotient (IQ) lower than 35.^2^ In 2007, there were 119 private and public institutions to care for people with SMID, and 72 national hospitals had special wards to ensure their care. It has been suggested that most people with SMID live at home with their families, but there are no data to confirm this.^1^ The approximate numbers of people with SMID in private/public institutions, national institutions, and homes in the community were estimated to be 5000, 7500, and 17 500 in 1980; 8500, 7500, and 29 000 in 1997; and 11 000, 7500, and 27 500 in 2003.^1^

A number of studies have shown that people with SMID are subject to various health problems and that they need appropriate medical care. However, information on the prognosis for survival is limited because formulating such estimates requires the resources of a large study. In most people with SMID, disability is diagnosed in infancy. Therefore, the prognosis for survival is an important concern for their family and for health care practitioners. In Japan, the Association of Welfare for Persons with Severe Motor and Intellectual Disability annually collects anonymous records of individual registrations and deaths of people with SMID from all private and public institutions, except national institutions. These data encompass 40% to 60% of people with SMID residing in institutions in Japan. Based on these data, we were able to estimate the prognosis for survival in people with SMID who had lived in a public or private institution in Japan between 1961 and 2003.

## METHODS

### Data source

The source of the data used in this study was the records kept on individual registrations and deaths at each institution housing people with SMID. In 1979, the Association of Welfare for Persons with Severe Motor and Intellectual Disability started the annual collection of individual registration records from all private and public institutions. The records submitted to the association have no personally identifiable information. In 2007, a study group of the association examined 197 425 records submitted between 1979 and 2007 and identified 16 409 persons who had lived in one of 119 institutions between 1961 and 2007. The observation period of the present study was between 1961 and 2003; thus, people who entered institutions after January 1, 2004 were excluded from our analyses—14 516 remained for analysis. For the present study, we used the 2003 database on deaths, which had been submitted to the association. The database detailed all deaths from the year that each institution was established to the year 2003. Data on individual registrations and deaths were merged by personal ID and institutional ID. The following data were provided for this study: personal ID, institutional ID, date of birth, date of admission, date of leaving institution, date of final certification of survival, level of disability, cause of disability, reason for leaving institution, final score on Oshima’s classification, date of death, and cause of death. Cause of death was recorded using an original code table established by the study group. Individual records with missing data for date of birth, date of leaving institution, or date of final certification of survival were excluded from our analyses, after which the records of 14 509 persons remained for analysis. Of these 14 509 persons, 10 603 were born before the establishment of the institutions in which they were eventually to reside. Children with SMID are usually admitted to general hospitals after birth. After leaving hospitals, most live in their family’s home, and some are admitted to institutions. Some with particularly severe disabilities die before admission to institutions. Thus, because people with SMID who resided in an institution that was established after they were born might have greater than average longevity, they were excluded from our analyses, after which the records of 3906 persons remained for further analysis. In addition, only people with disability types 1 to 4, according to Oshima’s classification described above, were included in this study. Type 1 is used to describe people who are bedridden and have an IQ lower than 20; type 2 describes those who are able to sit, crawl, or walk with support and have an IQ lower than 20; type 3 describes those who are able to sit, crawl, or walk with support and have an IQ lower than 35; and Type 4 describes those who are bedridden and have an IQ lower than 35. Ultimately, data from 3221 people were analyzed: 2462 (63.0%) with type 1, 607 (15.5%) with type 2, 67 (1.7%) with type 3, and 85 (2.2%) with type 4 disability. We also performed subgroup analyses that excluded infants who died before the age of 2 years and persons whose observation period was 2 years or less, because they were usually cared for in general hospitals and might therefore have a very different prognosis for survival than that of people with SMID living in institutions. In addition, each institution decided whether data on infants were submitted to the association, which could have resulted in selection bias. When we excluded data from these infants, data from 3185 people remained for analysis: 2427 (62.7%) with type 1, 607 (15.5%) with type 2, 67 (1.7%) with type 3, and 84 (2.2%) with type 4 disability.

### Statistical analysis

For people who were identified as having died, life spans were calculated as the period from birth to death. For people who were still residing at the institution and alive at the time of this study, the follow-up period was considered as the period from birth to December 31, 2003. If an individual had left their institution before December 31, 2003, the censoring date was the date of final certification of survival. The original record of death or certification of survival indicated only the calendar year; thus, July 15 was defined as the censoring date. Kaplan–Meier survival estimates were then computed. Equality of survivor function between pairs of disability types, as determined according to Oshima’s classification, was evaluated by the log-rank test. A multiple comparison among the 4 groups was performed using Bonferroni’s method, ie, a *P* value less than 0.008 (0.05/6) was considered statistically significant. All calculations were performed using statistical software (STATA, StataCorp LP, College Station, Texas, USA).

## RESULTS

The records of 3221 people with SMID showed that 2645 were alive and 576 had died. Table 1 shows the status (ie, alive/dead/left institution) of the subjects at the end of the observation period, according to the type of disability. The proportion of subjects lost to follow-up was 27.6%, which comprised 27.9% of those with a type 1 disability, 23.9% with type 2, 41.8% with type 3, and 35.3% of those with type 4 disability. The difference in the rates of type 2 and type 3 subjects lost to follow-up was statistically significant on a multiple comparison test (chi-square test adjusted by Bonferroni’s method, *P* = 0.001). The mean and median follow-up periods of the study subjects were 17.5 (SD 9.6) and 16.7 years, respectively. The major causes of disability were hypoxic encephalopathy/postnatal distress in 20.0%, unknown prenatal causes in 10.7%, external causes including brain injury in 8.3%, meningitis/encephalitis in 7.0%, hydrocephalus in 4.7%, and epilepsy in 4.5%. Major causes of death were pneumonia/bronchitis in 27.4%, other respiratory diseases in 19.4%, cardiac failure in 13.5%, other cardiovascular diseases in 5.2%, unknown causes in 5.0%, and sudden death in 4.3%. Before 1990, the major causes for death were pneumonia/bronchitis in 33.9%, cardiac failure in 16.1%, other respiratory diseases in 9.8%, unknown causes in 6.3%, other cardiovascular diseases in 5.4%, and seizure in 5.4%. After 1990, the major causes were pneumonia/bronchitis in 25.9%, other respiratory diseases in 16.0%, cardiac failure in 13.0%, other cardiovascular diseases in 4.7%, sudden death in 4.7%, and unknown causes in 4.7%. Only 1.1% of deaths after 1990 were due to seizure.

**Table 1.** Status of subjects according to disability type

Status	Type of disability				Total

	1	2	3	4	
Alive at end of study period	1254	425	34	42	1755
Dead	521	37	5	13	576
Left institution	687	145	28	30	890
Total	2462	607	67	85	3221

The details of type 1–4 disability are described in the text.

Table 2 shows the survival rates at ages 10, 20, 30, and 40 years. The survival rate at age 20 years for all subjects was 79% (95% confidence interval [CI], 78%–81%). People with type 1 disability had the lowest survival rates at all ages. The log-rank test showed that type 1 disability was associated with a significantly lower survival rate than type 2 (*P* < 0.0001). Type 1 disability was also associated with a lower survival rate than types 3 and 4, but the differences were not significant (*P* = 0.01, 0.03, respectively). Type 4 disability was associated with a significantly lower survival rate than type 2 (*P* = 0.001). People who were unable to sit had lower survival rates than those who could. Among people who could not sit, those with lower IQs had a lower survival rate. As described in the Methods section, we performed subgroup analyses that excluded infants who died before the age of 2 years and persons whose observation period was 2 years or less. The results did not substantially differ from those of the general analysis.

**Table 2.** Survival rate for people with severe motor and intellectual disabilities living in 119 private and public institutions in Japan (1961–2003)

Type of disability	Survival rate (95% confidence interval)			

	Age 10	Age 20	Age 30	Age 40
Including all deaths and observation period ≥0 years				
Types 1–4	0.91 (0.90, 0.92)	0.79 (0.78, 0.81)	0.72 (0.70, 0.74)	0.64 (0.59, 0.69)
Type 1	0.87 (0.86, 0.89)	0.73 (0.70, 0.75)	0.64 (0.61, 0.67)	0.58 (0.52, 0.64)
Type 2	0.98 (0.96, 0.99)	0.95 (0.92, 0.96)	0.91 (0.87, 0.94)	0.86 (0.76, 0.92)
Type 3	0.94 (0.82, 0.98)	0.88 (0.74, 0.95)	0.88 (0.74, 0.95)	NA
Type 4	0.97 (0.90, 0.99)	0.87 (0.75, 0.93)	0.77 (0.60, 0.87)	0.52 (0.18, 0.78)

Excluding death at age ≤2 years or observation period ≤2 years				
Types 1–4	0.91 (0.90, 0.92)	0.78 (0.77, 0.80)	0.72 (0.69, 0.74)	0.65 (0.59, 0.69)
Type 1	0.88 (0.86, 0.89)	0.72 (0.71, 0.75)	0.64 (0.61, 0.67)	0.58 (0.52, 0.64)
Type 2	0.98 (0.96, 0.99)	0.95 (0.92, 0.96)	0.91 (0.87, 0.94)	0.86 (0.76, 0.92)
Type 3	0.94 (0.82, 0.98)	0.88 (0.74, 0.95)	0.88 (0.74, 0.95)	NA
Type 4	0.97 (0.90, 0.99)	0.87 (0.75, 0.93)	0.77 (0.60, 0.87)	0.52 (0.18, 0.78)

NA: data not available.

The details of type 1–4 disability are described in the text.

Table 3 shows survival rates according to birth year range. At age 10 years, people born after 1990 had a significantly lower survival rate than did those born before 1990. At age 20 years, people born after 1980 had a significantly lower survival rate than did those born before 1980. The estimates for people with type 1 disability were similar to the overall estimates.

**Table 3.** Survival rate for people with severe motor and intellectual disabilities living in 119 private and public institutions in Japan (1961–2003), according to birth year range

Birth date/type of disability	Survival rate (95% confidence interval)			

	Age 10	Age 20	Age 30	Age 40
Before Dec. 31, 1969				
Types 1–4	NA	0.89 (0.80, 0.95)	0.82 (0.72, 0.89)	0.74 (0.62, 0.82)
Type 1	NA	0.84 (0.70, 0.92)	0.75 (0.60, 0.85)	0.66 (0.50, 0.78)

Jan. 1, 1970–Dec. 31, 1979				
Types 1–4	0.95 (0.93, 0.96)	0.83 (0.80, 0.86)	0.76 (0.73, 0.79)	NA
Type 1	0.94 (0.91, 0.95)	0.77 (0.73, 0.80)	0.69 (0.67, 0.73)	NA

Jan. 1, 1980–Dec. 31, 1989				
Types 1–4	0.88 (0.86, 0.90)	0.76 (0.73, 0.78)	NA	NA
Type 1	0.85 (0.83, 0.87)	0.71 (0.68, 0.74)	NA	NA

After Jan. 1, 1990				
Types 1–4	0.86 (0.82, 0.88)	NA	NA	NA
Type 1	0.84 (0.81, 0.87)	NA	NA	NA

Excluding deaths at 2 years of age or younger or an observation period of ≤2 years.

NA: data not available.

The details of type 1–4 disability are described in the text.

## DISCUSSION

We estimated the prognosis for survival among people with SMID living in public and private health care facilities in Japan. As expected, their survival rate was much worse than that of the general population, and people with more severe physical disability had worse prognoses. Sitting function was an import factor for prognosis, a finding that is in complete agreement with previous reports.^1^^,^^3^ In addition, we speculate that severe cognitive disability affects prognosis, along with mobility. The survival rate decreased between 1958 and 2003, most likely because people with more severe disabilities have been admitted recently to institutions, after medical treatment in the newborn intensive care units (NICU) of general hospitals.^1^

In this study, the severity of disability was assessed by using Oshima’s classification, which was established for administrative purposes and consists of evaluations of mobility and IQ. Disability type is assessed for all people with SMID in institutions. Disability type can change and is therefore reassessed annually. We used the final assessment of disability type because we suspected that this assessment was most closely linked to survival.

There are few data on the prognosis for survival in people with SMID in Japan. In 1996, the survival rate at 10 years was reported to be 63%, based on data from 183 people with SMID living with their family in the Tokyo metropolitan area.^4^ In national institutions, the survival rate at 8 years was 75% to 95% between 1988 and 1995, and higher mortality was observed at younger ages.^4^ International comparison of survival rates is difficult because the SMID classification is unique to Japan. Concerning the prognosis for survival in people with cerebral palsy, a recent study found that those with more severe disabilities had a lower survival rate, which accords with our estimates.^5^ The people included in the present study encompassed 40% to 60% of people with SMID who stay in institutions in Japan. Therefore, we believe that they are representative of people with SMID in institutions, although their characteristics might differ from those of people with SMID who stay in national institutions.

The prognosis for survival differed between people living with their family and those living in institutions. Previous studies in Japan have reported that people with SMID living in the community had worse prognoses than those living in national institutions.^4^ The reasons for admission to institutions are not limited to physical status; the presence of vacancies in an institution and the family’s socioeconomic status are also important. We believe that there are differences in the quality of health care, family socioeconomic status, and mental status of people with SMID living with their family and those living in institutions. Regarding people with cognitive disabilities, mortality is believed to be higher among those living in institutions, as compared to those living in the community.^6^

An important question is whether intellectual disability affects survival prognosis. In this study, among people who could not sit, those with lower IQs had a lower survival rate. However, physical disability was a more important factor for survival.

In epidemiological studies of cerebral palsy, misclassification is a possibility, because cerebral palsy is a syndrome rather than a disease.^5^ Although SMID is a complex of conditions, the definition of SMID is straightforward and should not lead to significant misclassification.

In order to avoid selection bias with respect to longevity, we excluded people who were born before the establishment of the institutions in which they were eventually to reside. We also performed subgroup analyses that excluded infants who died before the age of 2 years and persons whose observation period was 2 years or less; the findings did not substantially differ from those of the general analysis. Mean age at admission was 8.8 years; thus, infants with more severe disability were excluded from analyses in this study.

The proportion lost to follow-up was 28%. It is not uncommon for people with SMID to move to other institutions and hospitals, or to go home to live with their family. People who moved to other private or public institutions were registered at the new institutions. The proportion lost to follow-up was high among people with type 3 disability, probably because type 3 disability is the least severe, and thus allowed them more opportunity to relocate.

This study did have some limitations. First, although the source data included annual records of people with SMID in institutions, and all institutions sent data on all their residents, 3538 of the 197 425 (1.8%) records submitted did not indicate the personal and/or institutional ID of the potential subject. Because each person has a number of annual documents, it is impossible to determine how many eligible persons were missing from the records. Secondly, the number of elderly people was small because the follow-up periods were relatively short. Therefore, survival rates at advanced ages could not be estimated or had very wide confidence intervals.

The major causes of death were respiratory diseases and cardiovascular diseases, which confirms the findings of earlier reports.^1^^,^^3^ The proportions of deaths attributable to these 2 disease categories did not change within the study period, although the proportion of deaths due to pneumonia/bronchitis decreased after 1990. This finding was in accord with a previous report.^3^ We observed that the proportion of deaths due to seizure decreased after 1990, most likely due to improved medical care for seizure and better control of epilepsy. In the future, we expect the major causes of death in this cohort to change with aging.

Epilepsy, feeding disorders, and respiratory disorder are possible risk factors for death. However, these factors were not assessed in this study because the relevant information was not available. Epilepsy is observed in 50% to 70% of people with SMID, and is often difficult to cure.^1^ Epilepsy was not a major cause of death in this study, but it may have had an effect on functional disability. Feeding and swallowing disorders are often observed in people with SMID, and frequently lead to aspiration pneumonia. Tube feeding, gastrostomy, and jejunostomy are widely used, but the effects of these treatments on the prognosis for survival is unknown. Positioning of these treatments varies depending on the clinical assessment of each case.^5^ It is unclear whether these treatments were risk prediction factors (ie, people with more severe disabilities, and hence a worse prognosis, required these treatments) or risk reduction factors (ie, the treatments improved prognosis). Therefore, although there might be a relation between these treatments and prognosis for survival, it is difficult to clarify whether the relation is positive or negative. Studies of people with cerebral palsy have yielded controversial results regarding whether gastrostomy feeding increases the risk of death.^7^^,^^8^ In the present study, most deaths were due to respiratory diseases. A previous study also suggested that many more deaths are caused by respiratory disorders in patients with cerebral palsy.^9^ Consequently, the presence of respiratory disorders could affect prognosis for survival. In future studies, these factors should be investigated as possible coexisting factors related to prognosis.

In conclusion, the prognosis for survival of people with SMID living in public and private institutions in Japan has not improved since the 1960s and is much worse than that of the general population. We also found that sitting function was an important factor for prognosis. Finally, although intellectual disability might affect prognosis, morbidity was a more important factor.
